# Comparative Analysis of the Complete Plastid Genome of Five *Bupleurum* Species and New Insights into DNA Barcoding and Phylogenetic Relationship

**DOI:** 10.3390/plants9040543

**Published:** 2020-04-22

**Authors:** Jun Li, Deng-Feng Xie, Xian-Lin Guo, Zhen-Ying Zheng, Xing-Jin He, Song-Dong Zhou

**Affiliations:** Key Laboratory of Bio-Resources and Eco-Environment of Ministry of Education, College of Life Sciences, Sichuan University, Chengdu 610065, China; lijun_cq512@163.com (J.L.); df_xie2017@163.com (D.-F.X.); gxl759967@163.com (X.-L.G.); zoezhengzhenying@163.com (Z.-Y.Z.)

**Keywords:** Apiaceae, *Bupleurum*, plastid genome, comparative analysis, phylogeny, DNA barcoding

## Abstract

*Bupleurum* L. (Apiaceae) is a perennial and herbal genus, most species of which have high medicinal value. However, few studies have been performed using plastome data in this genus, and the phylogenetic relationships have always been controversial. In this study, the plastid genomes of *Bupleurum chinense* and *Bupleurum commelynoideum* were sequenced, and their gene content, order, and structure were counted and analyzed. The only three published *Bupleurum* species (*B. boissieuanum*, *B. falcatum,* and *B. latissimum*) and other fifteen allied species were selected to conduct a series of comparative and phylogenetic analyses. The genomes of *B. chinense* and *B. commelynoideum* were 155,869 and 155,629 bp in length, respectively, both of which had a typical quadripartite structure. The genome length, structure, guanine and cytosine (GC) content, and gene distribution were highly similar to the other three *Bupleurum* species. The five *Bupleurum* species had nearly the same codon usages, and eight regions (*petN-psbM*, *rbcL-accD*, *ccsA-ndhD*, *trnK(UUU)-rps16*, *rpl32-trnL(UAG)-ccsA*, *petA-psbJ*, *ndhF-rpl32,* and *trnP(UGG)-psaJ-rpl33*) were found to possess relatively higher nucleotide diversity, which may be the promising DNA barcodes in *Bupleurum*. Phylogenetic analysis revealed that all *Bupleurum* species clustered into a monophyletic clade with high bootstrap support and diverged after the *Chamaesium* clade. Overall, our study provides new insights into DNA barcoding and phylogenetic relationship between *Bupleurum* and its related genera, and will facilitate the population genomics, conservation genetics, and phylogenetics of *Bupleurum* in Apiaceae.

## 1. Introduction

*Bupleurum* L. (Apiaceae) is a large genus in Apiaceae, most species of which are perennial herbs. The genus contains about 180 species widely distributed in temperate and subtropical of the northern hemisphere, with 42 species (22 endemics) in China [1]. Most *Bupleurum* plants have high medicinal value and are widely used as a traditional medicine in Asia, Europe, and northern Africa [2]. *Bupleurum chinense* and *Bupleurum scorzonerifolium* are used as bupleuri radix to treat cold, chills and fever alternate, chest coerces bloated pain, etc. [3]. There are many studies focused on chemical components [4,5] and pharmacognosy [6,7], but those involved in the systematics analyses are relatively few. Meanwhile, the mixed usage of different *Bupleurum* species may bring adverse reactions due to the unawareness of the chemical component [8,9]. Molecular phylogenetic studies based on nuclear ribosomal internal transcribed spacer (nrITS) and plastid DNA introns (*rpl16* and *rps16*) supported a basal position of the genus *Bupleurum* within subfamily Apioideae, and considered this genus a distinct tribe [10,11]. However, the phylogenetic relationships within *Bupleurum* have always been controversial. Studies based on morphology, chromosome counts, nrDNA ITS sequences, and plastid DNA markers laid the theoretical basis for the speciation and phylogenetic relationship analyses among *Bupleurum* species [12,13,14]. However, there still needs to be more molecular information to determine the interspecific phylogeny more accurately. In recent years, comparative analysis of the complete plastid genome has become a promising method for population genetics, conservation genetics and phylogenetic studies [15,16]. However, only three *Bupleurum* species’ complete plastid genomes (*Bupleurum boissieuanum*, *Bupleurum falcatum,* and *Bupleurum latissimum*) have been reported until now [17,18].

The typical plastid genome in angiosperms is a circular molecule of double-stranded DNA, which ranges from 120 kb to 170 kb in length and usually encodes 120 to 130 genes [19]. Typical plastid genomes have a quadripartite structure consisting of a small single-copy region (SSC) and a large single-copy (LSC) region jointed by a pair of inverted repeats (IRa and IRb). The gene content, order and structure are highly conserved at low taxonomic levels [20], but there are still variations especially in the intergenic regions and IR boundaries. Some hotspot regions with much nucleotide information have been applied to species identification [21,22]. The evolutionary rates of conserved coding regions are low, which are high in non-coding regions. The former is suitable for phylogenetic studies of high taxonomic levels such as orders and families, and the latter is suitable for taxa that differentiated recently. Moreover, the genetic pattern of plastid is matrilineal, thus making plastid DNA easier to track individual lineages in time and space than nuclear DNA that constantly merge and recombine genes from two parents [23]. Compared with nuclear genomes, plastid genomes are small but contain a lot of information, and they are easier to sequence. Due to these advantages, plastid genomes have been widely applied to molecular identification, divergence dating and phylogenetic analysis [20,24,25,26,27].

The rapid development of high-throughput sequencing technology has greatly facilitated the acquisition of genome data. With the advantages of high-throughput and low cost-effectiveness, an increasing number of plastid genomes of plants have been sequenced and assembled in recent years, and more genetic resources are analyzed via different bioinformatics approaches [28,29]. The high-throughput sequencing technology and vast information of plastid genomes mark a new era of population genomics and phylogenetic studies.

In our study, we used the NGS to obtain the complete plastid DNA sequences of two *Bupleurum* species (*B. chinense* and *B. commelynoideum*), then counted and analyzed their gene content, order and structure. We also combined the only three published *Bupleurum* species (*B. boissieuanum*, *B. falcatum* and *B. latissimum*) and allied plastid genome sequences to perform a series of comparative analyses including codon usage bias, repetitive sequences, nucleotide diversity, selective pressure and IR boundary comparative analysis, also phylogenetic analyses were conducted to infer their relationship. Our findings provide new insights into DNA barcoding and phylogenetic relationships of the genus *Bupleurum*, and will provide genetic resources for population genomics, conservation genetics and phylogenetics of *Bupleurum* in Apiaceae.

## 2. Results and Discussion

### 2.1. Genome Features of B. chinense and B. commelynoideum

**Figure 1:**
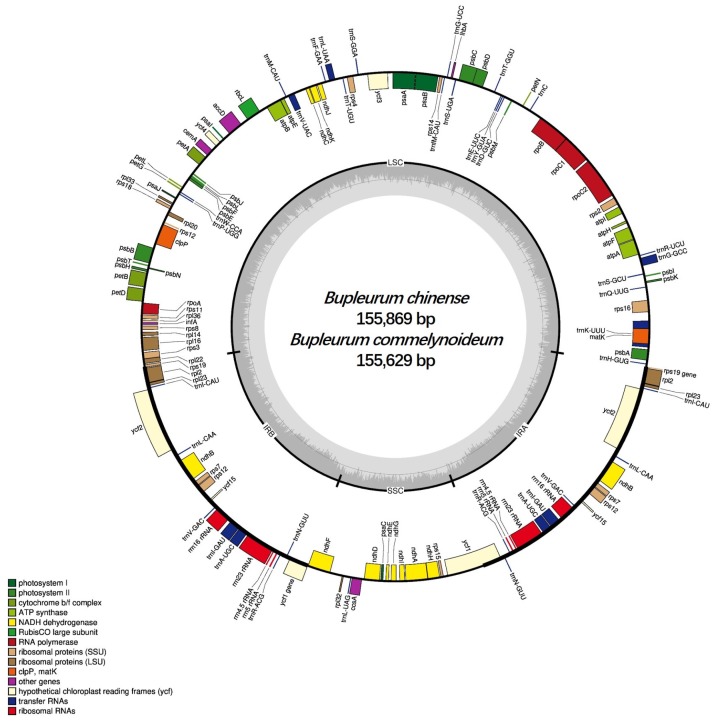
Plastid genome map of *B. chinense* and *B. commelynoideum*. Genes shown outside the outer circle are transcribed counterclockwise, while genes inside are transcribed clockwise. The colored bars indicate different functional groups. The darker gray area in the inner circle denotes GC content, while the lighter gray corresponds to AT content of the genome. LSC: large single-copy region; SSC: small single-copy region; IR: inverted repeat region.

The sequences of *B. chinense* and *B. commelynoideum* were 155,869 bp and 155,629 bp, respectively, both of which were found to have a typical quadripartite structure comprising a small single-copy region (SSC) and a large single-copy (LSC) region jointed by a pair of inverted repeats (IRa and IRb) (Figure 1). This was a conserved structure in most plastid genomes of higher plants [30,31]. The two plastid genomes showed the same overall GC content, which was 37.7%, and the same values of the LSC, SSC and IR regions which were 35.8%, 31.4%, and 42.8%, respectively, near to other three *Bupleurum* species (*B. boissieuanum, B. falcatum* and *B. latissimum*) plastid genome levels [17,18]. Like other species, GC contents in IRs are higher than in other regions, and this might result from rRNAs with high GC in the IRs [32,33,34]. Some other related data were very close to the other three species in *Bupleurum* (Table 1).

Both of the *B. chinense* and *B. commelynoideum* plastid genome contained 114 unique genes (Table 2) including 80 protein-coding genes (PCGs), 30 transfer RNA genes (tRNAs) and 4 ribosomal RNA genes (rRNAs). The SSC region contained 11 PCGs (*ndhF*, *rpl32*, *ccsA*, *ndhD*, *psaC*, *ndhE*, *ndhG*, *ndhI*, *ndhA*, *ndhH,* and *rps15*) and 1 tRNA (*trnL-UAG*), while the LSC region contained 60 PCGs and 22 tRNAs. 17 genes were duplicated in the IR regions, including 7 tRNAs (*trnA-UGC, trnI-CAU, trnI-GAU, trnL-CAA, trnN-GUU, trnR-ACG,* and *trnV-GAC*), 6 PCGs (*rps7, rpl2, rpl23, ndhB, ycf2* and *ycf15*), and 4 rRNAs (*rrn4.5*, *rrn5*, *rrn16* and *rrn23*). The genes *rps12* and *rps19* straddled the LSC and IR region, while gene *ycf1* straddled the SSC and IR region. 15 genes (*trnA-UGC, trnG-GCC, trnI-GAU, trnK-UUU, trnL-UAA, trnV-UAC, rpoC1, rps16, rpl2, rpl16, ndhA, ndhB, petB, petD* and *atpF*) harbored a single intron, and 3 genes (*rps12, ycf3* and *clpP*) contained two introns. In our study, the *infA*, *ycf15* and the incomplete copy of *ycf1* and *rps19* in the IR regions were regarded as pseudogenes. The gene *ycf68* was lost in the five *Bupleurum* species and most Apiaceae species [17,18,35,36,37], but existed in other families and was identified as a pseudogene [38,39]. Pseudogenization and pseudogene loss occurred in different plant taxa, and this may be caused by multiple genetic lesions and transfer to the nucleus [40,41].

### 2.2. Codon Usage Bias Analysis

**Figure 2:**
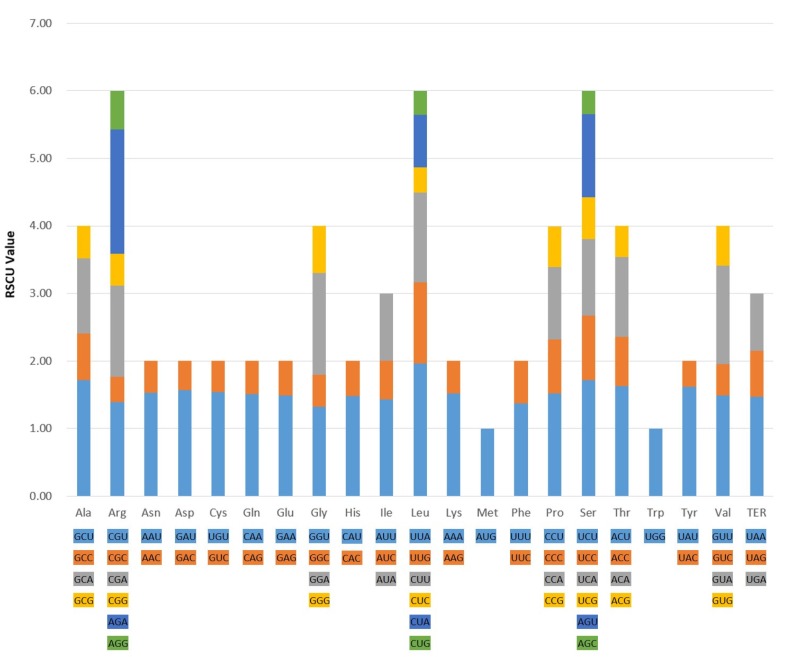
Codon content of 20 amino acids and stop codons in the five *Bupleurum* species.

In the plastid genomes of the five *Bupleurum* species (*B. chinense*, *B. commelynoideum*, *B. boissieuanum, B. falcatum,* and *B. latissimum*), the 20 amino acids were also encoded by 64 codons (Figure 2), among which only methionine (Met) and tryptophan (Trp) were encoded by single codon, while arginine (Arg), leucine (Leu) and serine (Ser) had the maximum codons of six. Most of the amino acids had codon preferences except Met and Trp. The total number of codons in the five *Bupleurum* species ranged from 21,188 to 21,195 (Table S1 in Supplementary Material). Leucine (Leu) and cysteine (Cys) were the most and least abundant amino acids, respectively. The relative synonymous codon usage (RSCU) values of the same codon were subequal, with a maximum difference of 0.2 for very few. 30 codons preferences were identified, including 24 high preference (RSCU > 1.3), 2 moderate preference (1.2 ≤ RSCU ≤ 1.3) and 4 low preference (1.0 < RSCU < 1.2). These codons were from 18 amino acids and 1 stop codon. Preferences between codons may result from mutation, selection, and random genetic drift, and be affected by translation efficiency, which may be an adaptive factor [42,43]. The ENC, CAI, CBI and FOP values ranged from 49.83 to 49.90, 0.166 to 0.167, −0.102 to −0.100 and 0.353 to 0.354, respectively, which meant there was no obvious preference in the five *Bupleurum* species’ plastid genomes (Table 3). All the GC3 content values were 0.269%, indicating that these genes preferred the codons ended with A/T, which is a universal phenomenon in the plastid genome of higher plants [44,45,46].

**Figure 3:**
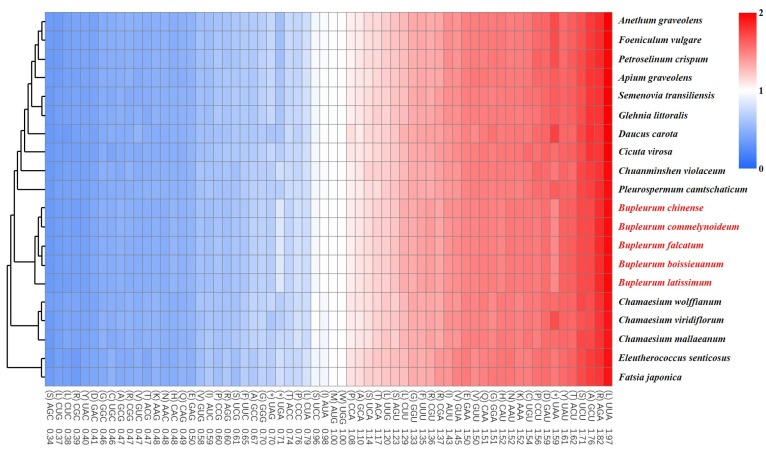
The codon distribution of the allied species in Apiaceae and outgroups. Red and blue indicate higher and lower RSCU values, respectively. The left of the figure shows the phylogenetic relationship among species. *Eleutherococcus senticosus* and *Fatsia japonica* are the outgroups from Araliaceae.

The five *Bupleurum* species and their allied species in Apiaceae had similar codon usages, of which UAA, AGA, GCU, UCU and ACU had the highest frequency, while AGC, CUG, CUC, CGC and UAC had the lowest frequency (Figure 3). This indicated that codons preferred to end with A/T, which was consistent with the conclusion of GC3 content above. Notably, the five *Bupleurum* species exhibited lower usages in the stop codon UAA (light red) and higher usages in the stop codon UGA (light blue). On the contrary, *Daucus carota*, *Pleurospermum camtschaticum* and *Chamaesium viridiflorum* showed higher usages in UAA (deep red) and lower usages in UGA (deep blue). These differences in the use of stop codons suggest that stop codons may not undergo the same strict selection as other codons. In addition, the codon CGU, CUU, and CAC had slightly higher usages, and CGA, AGG, UUC, and AUC had slightly lower usages than other allied species. The synonymous codons are generated by mutations, and the evolutionary pressures cause the use of these synonymous codons to vary in frequency [42,47]. Codon preference is the result of long-term adaption of species to their base composition, tRNA abundance, and environmental selection pressure. Moreover, the preference can affect the initiation, elongation, and accuracy of the translation, the shearing of mRNA, and the folding of proteins [48,49,50]. These preferences will contribute to the plastid gene engineering of *Bupleurum* species and lay a theoretical foundation for modification and efficient expression of exogenous genes.

### 2.3. Repetitive Sequences Analysis

**Figure 4:**
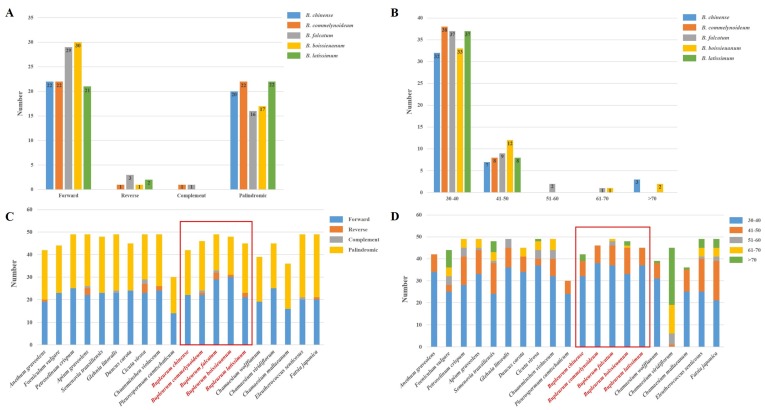
Analysis of short dispersed repeats (SDRs) in the five *Bupleurum* and allied species. (**A**) Numbers of four types SDRs in *Bupleurum.* (**B**) Numbers of different lengths of SDRs in *Bupleurum*. (**C**) Comparison of SDR types of allied species. (**D**) Comparison of SDR lengths of allied species. The five *Bupleurum* species were framed red.

Repetitive sequences in plastid genomes play an essential role in population genetics and biogeography studies [51,52], and these repeats may result from slipped-strand mispairing and improper recombination [53]. In this study, short dispersed repeats (SDRs) analysis found 22 forward, 0 reverse, 0 complement and 20 palindromic repeats in the plastid genome of *B. chinense* (Figure 4A), of which 32 were 30–40 bp in length, 7 were 41–50 bp in length, and 3 exceeded 70 bp in length (Figure 4B). Similarly, 22 forward, 1 reverse, 1 complement and 22 palindromic repeats were found in *B. commelynoideum*, of which 38 were 30–40 bp in length and 8 were 41–50 bp in length. Together with the other three *Bupleurum* species (*B. boissieuanum, B. falcatum,* and *B. latissimum*), they tended to generate more forward and palindromic repeats rather than reverse and complement repeats (Figure 4C). Comparing to the repeats with length more than 50 bp, the repeats with 30–40 bp in length more widely existed in the plastid genomes (Figure 4D). Additionally, repeat numbers are also obviously different. All the tendencies were the same in the allied species of the Apiaceae except that *Chamaesium viridiflorum* had a reversed length distribution in SDRs (Figure 4D). To figure out whether the abnormal SDRs distribution appears in a single species, a population or the whole genus, more comparative analysis needs to be performed on different levels in the future.

**Figure 5:**
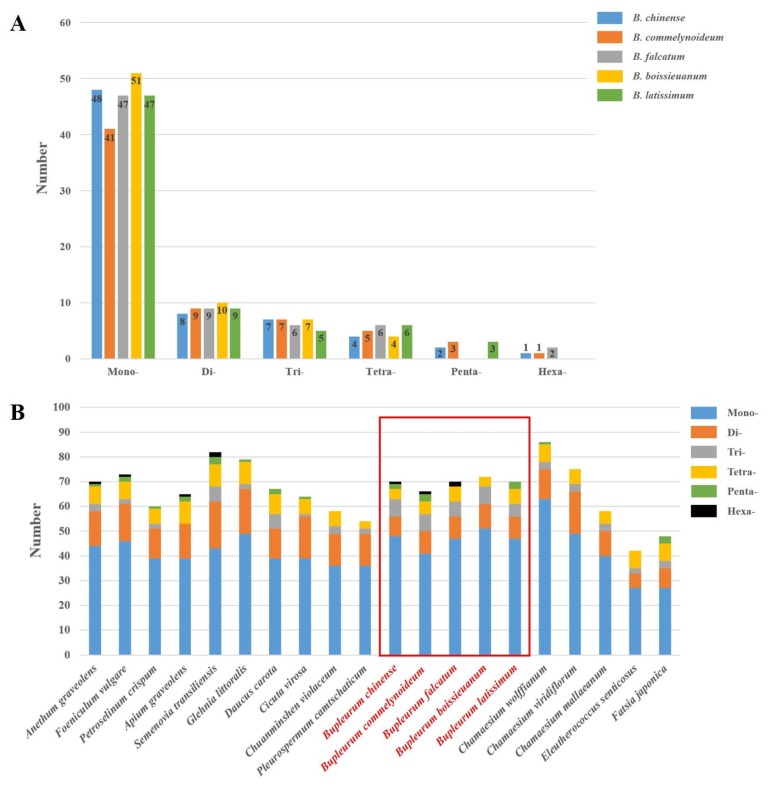
Analysis of simple sequence repeats (SSRs) in the five *Bupleurum* and allied species. (**A**) Numbers of six types SSRs in *Bupleurum*. (**B**) Comparison of SSR types of allied species. The five *Bupleurum* species were framed red.

Simple sequence repeats (SSRs) analysis showed 48 mono-, 8 di-, 7 tri-, 4 tetra-, 2 penta- and 1 hexa-nucleotides in *B. chinense*, and the corresponding values of *B. commelynoideum* were 41, 9, 7, 5, 3 and 1, respectively (Figure 5A). Among these sorts of SSRs, the number of mono-nucleotides was largest, and the number of di-, tri- and tetra-nucleotides were far less than the mono-nucleotides. Penta- and hexa-nucleotides had the least number, even some species existed no penta- and hexa-nucleotides. This tendency was also found in allied species (Figure 5B). Furthermore, the size distribution of SSRs varies in different species. In most species such as *Lilium* [54], *Primula* [55], *Allium* [56] and *Quercus* [57], the most abundant repeats are the mono-nucleotide repeats, but in *Forthysia* [58] are di-nucleotide repeats, and in *Nitotiana* [59] are tri-nucleotide repeats. This indicates that SSR variations will devote to genetic diversity in different species. Previous studies suggested that repeats diversity in plastid genomes are highly related in the rearrangement of the genome, and are generated by slipped-strand mispairing and abnormal recombination [53,60] in the process of DNA replication. Thus, SSRs have been widely used as molecular markers in population genetics and evolutionary studies [61,62].

### 2.4. Nucleotide Diversity Analysis

**Figure 6:**
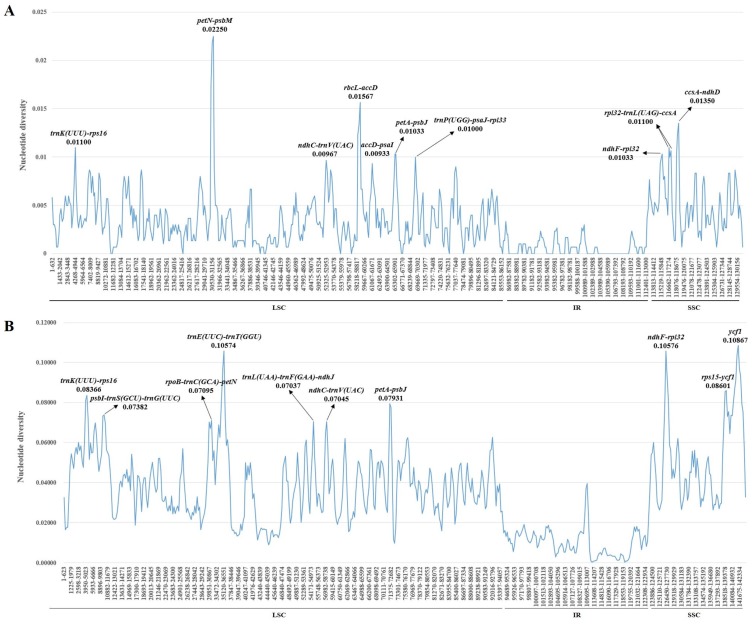
The nucleotide diversity of the plastid genome of (**A**) the 5 *Bupleurum* species and (**B**) 18 allied species in Apiaceae. Ten regions with the highest Pi values were marked out. LSC: large single-copy region; IR: inverted repeats region; SSC: small single-copy region.

Nucleotide diversity (Pi) of the plastid genomes in the five *Bupleurum* species was calculated to assess the sequence divergence level in the genus *Bupleurum*. In the LSC region, Pi values ranged from 0 to 0.02250, with an average of 0.00358, and in the SSC region, they ranged from 0.00067 to 0.01350, with an average value of 0.00505, while the IR region had the least average value of 0.00059, of which the Pi values ranged from 0 to only 0.00467 (Figure 6A). Low Pi values in the IR region indicated that the IR region existed fewer mutations and was highly conserved at the genus level. Sequences with high Pi values were all spacer regions between genes. Among these spacer regions, *petN-psbM*, *rbcL-accD*, *ccsA-ndhD*, *trnK(UUU)-rps16*, *rpl32-trnL(UAG)-ccsA*, *petA-psbJ*, *ndhF-rpl32,* and *trnP(UGG)-psaJ-rpl33* were the only eight regions which had >0.01000 Pi values. Intergenic regions are under weaker selection pressure and possess a higher evolutionary rate than genes. They are more suitable for the study of the classification and evolution of low taxonomic levels [22,63]. Most of the *Bupleurum* species have high medicine values, such as *B chinense* and B. *scorzonerifolium* [2], so identifying these species becomes important and necessary. Therefore, these eight hotspot sequences may be the promising DNA barcodes for identification, classification and genetic divergence of the *Bupleurum* taxa, and some of these have been used in other species [64].

Also, Pi values together within the other 13 allied species were calculated, and the same tendency was observed over the Pi variations (Figure 6B). The Pi values of LSC, SSC and IR region ranged from 0.00897 to 0.10574, 0.02639 to 0.10867 and 0.00037 to 0.05375, respectively, and the corresponding averages were 0.03831, 0.05282 and 0.01058, respectively. The averages of LSC and SSC region were nearly ten times as large as those in the five *Bupleurum* species. Furthermore, more than half of the higher diversity regions were different from those in the five *Bupleurum* species, which indicated a great difference in the sequence differentiation among the genus. The three highest diversity regions were *ycf1*, *ndhF-rpl32* and *trnE(UUC)-trnT(GGU)*, and the Pi values were 0.10867, 0.10576 and 0.10574, respectively, which were the only three regions whose Pi values were over 0.10000. These regions are more suitable for the study of genus levels, and have been widely used as molecular markers in previous phylogenetic studies [65,66,67], and easier to align than nrITS. In addition, we chose the ten regions with the highest Pi values for the phylogenetic analysis.

### 2.5. Selective Pressure Analysis

**Figure 7:**
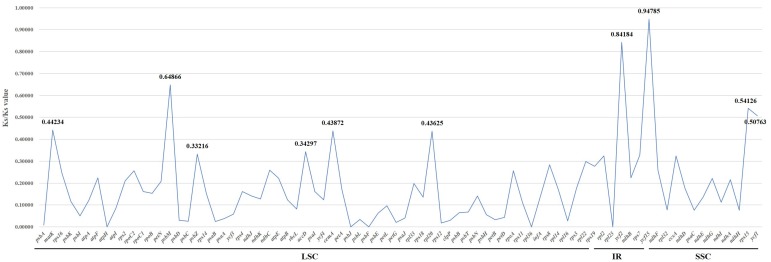
Selective pressure of 80 protein-coding genes in the 5 *Bupleurum* species and 13 allied species in Apiaceae. Ka: rate of non-synonymous substitution; Ks: rate of synonymous substitution.

The ω (Ka/Ks) of 80 protein-coding genes were calculated to assess the selection pressure among the five *Bupleurum* species and allied species in Apiaceae. Genes perform important biological functions, and the mutations will undergo rigorous selection. When the ratio of non-synonymous mutation rate (Ka) to synonymous mutation rate (Ks) is greater than 1, the gene is under positive selection. The Ka/Ks < 1 illustrates purifying selection and Ka/Ks close to 1 illustrates neutral evolution [68,69]. The result showed that there were no genes with Ka/Ks > 1, indicating that no genes were under positive selection (Figure 7). The gene *ycf15* had the highest Ka/Ks value of 0.94785, while the gene *ycf2* with the Ka/Ks value of 0.84184 ranked second. Both of them had values close to 1, which indicated that there could be as many non-synonymous mutations as synonymous mutations, and they might be in the process of neutral evolution, especially *ycf15*. However, the gene *ycf15* is regarded as a pseudogene that had lost its function (Table 2), which indicates that mutations will not undergo selection. Also, the gene *ycf2* is one of the Hypothetical Chloroplast Reading Frames (YCF) (Table 2), so it is controversial whether *ycf2* encodes a protein [70]. This may be the reason why *ycf15* and *ycf2* underwent a neutral evolution. The remaining genes with Ka/Ks away from 1 were regarded under purifying selection. We found the Ka values of *atpH*, *psbJ*, *psbF*, *rpl36* and *rpl*23 were 0, and thereby the Ka/Ks values were all 0 in Figure 7. Among them, genes *atpH*, *psbJ* and *psbF* are related to photosynthesis, and genes *rpl36* and *rpl23* are for the synthesis of large ribosome subunits (Table 2). All these genes are crucial to the plant, and non-synonymous mutations that occurred can affect survival and, thus, being eliminated.

### 2.6. IR Boundary Comparative Analysis

**Figure 8:**
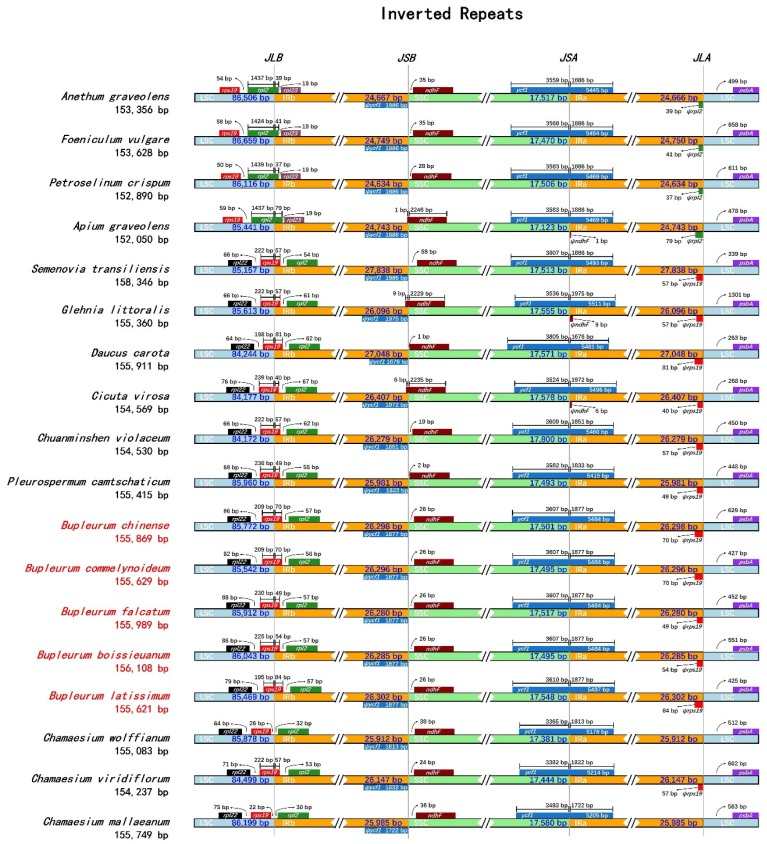
Comparison of the LSC, SSC and IR junction among the five *Bupleurum* and allied species plastid genomes. JLB: junction line between LSC and IRb; JSB: junction line between SSC and IRb; JSA: junction line between SSC and IRa; JLA: junction line between LSC and IRa.

The IR/LSC and IR/SSC junction of the five *Bupleurum* species and their allied species were compared to assess the expansion and contraction of the IR regions. Among the five *Bupleurum* species, the genes *rpl22* and *rpl2* flanked the LSC/IRb junction, and gene *rps19* traversed the LSC and the IRb region (JLB line), with 49–84 bp located in the IR region (Figure 8). Therefore, the portions of the *rps19* located in the IRb were also duplicated in the IRa/LSC junction, which was identified as pseudogenes (marked with ‘ψ’). The *ycf1* gene, which was the second-largest gene of the plastid genome in higher plants [30], traversed the SSC and IRa region, with the same length of 1877 bp in IR region, thus in the junction of the IRb/SSC lying the *ψycf1* with 1877 bp, too. On the other side of the IRb/SSC lied the gene *ndhF*, which was the same length of 26 bp away from the IRb/SSC junction. No obvious expansion or contraction was observed within the five *Bupleurum* species, but an obvious shift of JLB was observed in other species in Apiaceae, which indicated they were undergoing a contraction in IR regions. It’s worth noting that the gene *ndhF* traversed the IRb and SSC region, which indicated that there might also be a tendency of expansion in gene *ndhF.* Among Apiaceae species, the size variation of the plastid genome in the process of evolution mainly results from the expansion and contraction of the IR regions [71,72], and double-strand break (DSB) events may be the main reason of expansions [73].

### 2.7. Phylogenetic Analysis

**Figure 9:**
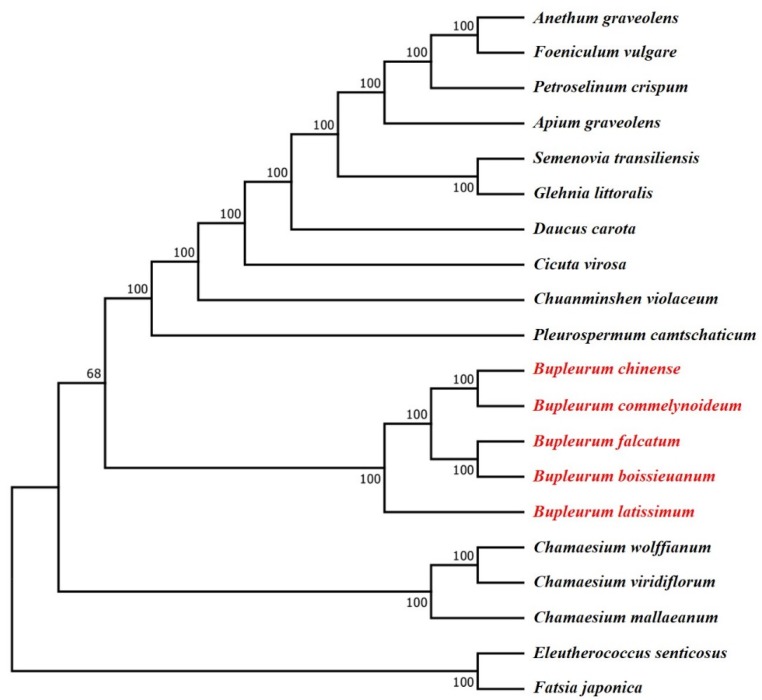
Molecular phylogenetic trees of 18 Apiaceae and 2 Araliaceae based on complete plastid genomes. The trees were constructed using maximum likelihood (ML) algorithm with GTR + G model and 1000 bootstrap replicates. The numbers above node are bootstrap support values.

Plastid genomes of plants have been widely used to investigate the phylogenetic and evolutionary relationships among families [27], genera [74], species [75], and even within species [76]. To investigate the phylogenetic position of the *B. chinense* and *B. commelynoideum* in Apiaceae, six partitions datasets including the complete plastid genomes, LSC regions, SSC regions, IR regions, single-copy CDS sequences and ten high-variation regions of 18 Apiaceae and 2 Araliaceae species plastid genomes were used to construct the maximum likelihood (ML) tree. All six datasets produced the same topology trees with a slight difference in bootstrap support (Figure S1). Among them, the trees based on SSC regions, single-copy CDS sequences and ten high-variation regions possessed higher bootstrap support values (>90), which suggested that these three datasets can better show species differentiation. All the phylogenetic trees revealed that *B. chinense* was most related to *B. commelynoideum*, and they were gathered within a clade (Figure 9). Also, *B. falcatum* and *B. boissieuanum* were gathered within a clade. These two clades were sister groups. *B. latissimum* was the first to speciate and was at the base among the five *Bupleurum* species in phylogenetic trees. The five *Bupleurum* species clustered into a monophyletic clade with strong support in all trees. The phylogenetic relationship in the genus *Bupleurum* was similar to previous studies based on nrITS and plastid DNA markers (*trnH-psbA* and *matK*) [14]. In our study, the *Bupleurum* clade was differentiated after the *Chamaesium* clade, and *Chamaesium* clade was at the base representing the basal taxa in Apioideae. However, in previous studies based on nrITS and plastid DNA markers, the *Bupleurum* clade was earlier differentiated than the *Chamaesium* and was at the base [10,11,77]. The inconsistent results indicated that the nrITS may be affected by hybridization and incomplete lineage sorting. Plastid DNA, which is maternally inherited, is longer and has less mutation than the nuclear ITS region, so it will provide more phylogenetic information and not be interfered with by paralogous genes in the phylogenetic studies [23,78]. Therefore, plastid DNA can better reflect the evolutionary relationship. This may be the main reason for the discrepancy in the phylogenetic analyses based on nrITS, plastid DNA markers, and plastid genomes. Anyway, the genus *Bupleurum* is still the relatively basal taxa in the Apiaceae. In addition, the genus *Daucus*, *Semenovia*, etc. are the crown groups, which is consistent with their external morphology and fruit characteristics [1,79]. Our study may provide information for population genomics and taxonomy in *Bupleurum*, and a new insight for phylogenetic reconstruction in Apiaceae.

## 3. Materials and Methods

### 3.1. Plant Materials and DNA Extraction

Mature and healthy leaves of single individuals of *B. chinense* and *B. commelynoideum* were collected from Mao county (Sichuan province, China; coordinates: 31°41′28.94″ N, 103°49′32.41″ E) and LuHuo county (Sichuan province, China; coordinates: 31°38′29 N, 100°15′05″ E), respectively. All voucher specimens were deposited in the Sichuan University Herbarium (SZ). The fresh leaves above were immediately dried with silica gel for further DNA extraction. The total genomic DNA was extracted from the dried leaves using the modified cetyltrimethyl ammonium bromide (CTAB) method [80].

### 3.2. Genome Sequencing, Assembly and Annotation

The total genomic DNA was sequenced at Novogene (Novogene BioTech, Inc. Beijing, China) by Illumina NovaSeq 6000 Platform (Illumina, San Diego, CA). Libraries with an average length of 350 bp were constructed, and the average length of generated reads was 150 bp. Deep coverage of plastid genomes was obtained from the total genomic DNA via genome skimming sequencing strategy [81]. The clean data were used to assemble the complete plastid genome via NOVOplasty [82] with the complete plastid genome of *B. boissieuanum* as the reference (GenBank accession No. MF663725). The assembled plastid genomes were annotated via Geneious v9.0.2 [83] with the sequence of *B. boissieuanum* as the reference, and the annotation result was revised according to the *B. latissimum*, *B. falcatum* and allied genus including *Angelica*, *Chamaesium*, *Chuanminshen,* and *Pleurospermum* manually. Finally, the physical maps of the genome were generated using OGDraw v1.3.1 [84] (http://ogdraw.mpimp-golm.mpg.de/). The annotated plastid genomes of *B. chinense* and *B. commelynoideum* had been submitted to GenBank under the Accession Number MN893666 and MT162552, respectively.

### 3.3. Codon Usage Bias Analysis

Codon usage analysis was conducted via the program codon W [85]. 53 protein-coding genes (CDS) (Table S2) were filtered from the five *Bupleurum* species (other three were downloaded from NCBI) after removing the CDS less than 300 bp and the repeat sequences. Five important indices were calculated to assess the extent of the codon usage bias including the codon adaptation index (CAI), codon bias index (CBI), frequency of optimal codons (Fop), GC content of the synonymous third codons positions (GC3s) and the effective number of codons (ENC). The relative synonymous codon usage values (RSCU) [86] of 15 allied species and 2 outgroup species (also downloaded from NCBI) were calculated to assess the difference of the codon usages (Table S3).

### 3.4. Repetitive Sequences Analysis

Short dispersed repeats (SDRs) were identified via REPuter [87], including forward, reverse, complement and palindromic. The parameters were set as follows: (1) Hamming distance of 3; (2) 90% or greater sequence identity; (3) minimal repeat size of 30 bp. Simple sequence repeats (SSRs) were also identified via perl script MISA [88], including mono-, di-, tri-, tetra-, penta- and hexa-nucleotides. The minimum numbers of the SSRs were set 10, 5, 4, 3, 3 and 3 for mono-, di-, tri-, tetra-, penta- and hexa-nucleotides, respectively.

### 3.5. Nucleotide Diversity Analysis

The plastid genome sequences were aligned using MAFFT [89] and adjusted manually. Then a slide window analysis was conducted to calculate the nucleotide diversity (Pi) in DnaSP v5 [90]. The parameters were set as follows: (1) windows size of 600 bp; (2) step size of 200 bp. Ten high-variation regions of the allied species were selected to conduct the phylogenetic analysis.

### 3.6. Selective Pressure Analysis

80 single-copy CDS sequences were extracted from the aligned plastid genome sequences after removing the repeat sequences. All the CDS sequences of the 18 species were aligned using MAFFT [89], then all the termination codons were removed. The final alignments were used to conduct the selective pressure analysis using DnaSP v5 [90]. The ratio (ω) of the non-synonymous substitution rate (Ka) to the synonymous substitution rate (Ks) was calculated to measure the selective pressure.

### 3.7. IR Boundary Comparative Analysis

All the plastid genome sequences were aligned using MAFFT [89] and adjusted manually. The plugin repeat finder in Geneious v9.0.2 was used to find the inverted repeats of some species without IR annotation. The genes and pseudogenes (marked with ‘ψ’) located in and beside the junctions of the boundaries were drawn manually to show the expansion and contraction of the IR region.

### 3.8. Phylogenetic Analysis

Phylogenetic analysis was conducted to investigate the relationship between the five *Bupleurum* species. Sixteen complete genome sequences of allied species were downloaded from NCBI, including other 3 species in *Bupleurum*, 3 species in *Chamaesium*, and 1 species in *Anethum, Foeniculum, Petroselinum, Apium, Semenovia, Glehnia, Daucus, Cicuta, Chuanminshen* and *Pleurospermum*. Two Araliaceae species (*Eleutherococcus senticosus* and *Fatsia japonica*) were added as outgroups (Table S3). The following six partitions datasets were used to conduct a phylogenetic analysis: (A) the complete plastid genomes; (B) the LSC regions; (C) the SSC regions; (D) the IR regions; (E) the single-copy CDS sequences; (F) ten high-variation region sequences. All the datasets were aligned and trimmed via MAFFT [89], and manually adjusted in MEGA7 if necessary. Maximum likelihood (ML) analysis was performed using RAxML v8.2.4 [91] with GTR + G model and 1000 bootstrap replicates.

## 4. Conclusions

In this study, we sequenced the complete plastid genomes of two *Bupleurum* species, *B. chinense* and *B. commelynoideum*, and compared their genome structure with the only three published *Bupleurum* species (*B. boissieuanum, B. falcatum,* and *B. latissimum*). All the five *Bupleurum* species plastid genomes exhibited a typical quadripartite and circular structure with very similar length, and the gene contents and orders were highly conserved. They also had very similar codon usage preferences but differed in two stop codon UAA and UGA from allied species. Repetitive sequences analysis showed they had the same tendency and certain diversity in numbers and distributions of SDRs and SSRs. The spacer regions *petN-psbM*, *rbcL-accD*, *ccsA-ndhD*, *trnK(UUU)-rps16*, *rpl32-trnL(UAG)-ccsA*, *petA-psbJ*, *ndhF-rpl32,* and *trnP(UGG)-psaJ-rpl33* had relative higher nucleotide diversity in five *Bupleurum* species, which could be the promising DNA barcodes in *Bupleurum* taxa, while *ycf1*, *ndhF-rpl32,* and *trnE(UUC)-trnT(GGU)* were the three highest regions with Pi values over 0.10000 compared with allied species, which have been widely used as molecular markers. No genes were under positive selection, but obvious shifts of JLB and traversing *ndhF* was observed in other species in Apiaceae. Phylogenetic analysis based on plastid genome datasets produced the same topology trees with high support. The five *Bupleurum* species clustered into a monophyletic clade and were later differentiated from the *Chamaesium* clade. This study will enrich the plastid genome data and genetic resources of the genus *Bupleurum* and provide new insights into DNA barcoding of *Bupleurum* and phylogenetic reconstruction of the family Apiaceae.

## Figures and Tables

**Table 1:** Summary of the genome features of the five *Bupleurum* species.

Region	*B. chinense*	*B. commelynoideum*	*B. falcatum*	*B. boissieuanum*	*B. latissimum*
Genome length (bp)	155,869	155,629	155,989	156,108	155,621
LSC length (bp)	85,772	85,542	85,912	86,007	85,471
SSC length (bp)	17,501	17,495	17,517	17,495	17,548
IR length (bp)	26,298	26,296	26,280	26,303	26,300
CDS length (bp)	78,249	78,255	78,261	78,270	78,264
Overall GC content (%)	37.7	37.7	37.7	37.7	37.6
LSC GC content (%)	35.8	35.8	35.8	35.8	35.8
SSC GC content (%)	31.4	31.4	31.4	31.4	31.3
IR GC content (%)	42.8	42.8	42.8	42.8	42.8
CDS GC content (%)	38.3	38.2	38.3	38.3	38.2

**Table 2:** List of genes present in *B. chinense* and *B. commelynoideum* plastid genome.

Groups	Categories	Name of Genes	Number
Self-replication	rRNAs	*rrn4.5*^(×2)^; *rrn5*^(×2)^; *rrn16*^(×2)^; *rrn23*^(×2)^	8
	tRNAs	*trnA-UGC*^(×2)^; *trnC-GCA*; *trnD-GUC*; *trnE-UUC*; *trnF-GAA*; *trnfM-CAU*; *trnG-GCC*; *trnG-UCC*; *trnH-GUG*; *trnI-CAU*^(×2)^; *trnI-GAU*^(×2)^; *trnK-UUU*; *trnL-CAA*^(×2)^; *trnL-UAA*; *trnL-UAG*; *trnM-CAU*; *trnN-GUU*^(×2)^; *trnP-UGG*; *trnQ-UUG*; *trnR-ACG*^(×2)^; *trnR-UCU*; *trnS-GCU*; *trnS-GGA*; *trnS-UGA*; *trnT-GGU*; *trnT-UGU*; *trnV-GAC*^(×2)^; *trnV-UAC*; *trnW-CCA*; *trnY-GUA*	37
	DNA-dependent RNA polymerase	*rpoA*; *rpoB*; *rpoC1*; *rpoC2*	4
	Small subunit of ribosomal proteins	*rps2*; *rps3*; *rps4*; *rps7*^(×2)^; *rps8*; *rps11*; *rps12*^(×2)^; *rps14*; *rps15*; *rps16*; *rps18*; *rps19*	14
	Large subunit of ribosomal proteins	*rpl2*^(×2)^; *rpl14*; *rpl16*; *rpl20*; *rpl22*; *rpl23*^(×2)^; *rpl3*; *rpl33*; *rpl36*	11
Genes for photosynthesis	Subunits of NADH dehydrogenase	*ndhA*; *ndhB*^(×2)^; *ndhC*; *ndhD*; *ndhE*; *ndhF*; *ndhG*; *ndhH*; *ndhI*; *ndhJ*; *ndhK*	12
	Subunits of photosystem Ⅰ	*psaA*; *psaB*; *psaC*; *psaI*; *psaJ*; *ycf3*; *ycf4*	7
	Subunits of photosystem Ⅱ	*psbA*; *psbB*; *psbC*; *psbD*; *psbE*; *psbF*; *psbH*; *psbI*; *psbJ*; *psbK*; *psbL*; *psbM*; *psbN*; *psbT*; *psbZ*	15
	Subunits of cytochrome b/f complex	*petA*; *petB*; *petD*; *petG*; *petL*; *petN*	6
	Subunits of ATP synthase	*atpA*; *atpB*; *atpE*; *atpF*; *atpH*; *atpI*	6
	Large subunit of rubisco	*rbcL*	1
Other genes	ATP-dependent protease subunit P	*clpP*	1
	Maturase	*matK*	1
	Subunits of Acetyl-CoA-carboxylase	*accD*	1
	Envelop membrane protein	*cemA*	1
	C-type cytochrome synthesis gene	*ccsA*	1
Genes of unknown function	Hypothetical chloroplast reading frames	*ycf1*; *ycf2*^(×2)^	3
	Pseudogenes	*infA*; *ycf15*; ycf1^*^; *rps19* *	4
Total		114 single-copy genes, 133 in total.	

^(×2)^ means the gene with two copies; * means the incomplete copy located in the IR of the gene straddling the IR and LSC/SSC regions.

**Table 3:** The indexes of the codon usage bias in the five *Bupleurum* species.

Index	*B. chinense*	*B. commelynoideum*	*B. falcatum*	*B. boissieuanum*	*B. latissimum*
Length (bp)	63,564	63,570	63,576	63,585	63,579
Codon No.	21,188	21,190	21,192	21,195	21,193
Amino acid No.	21,135	21,137	21,139	21,142	21,140
SC No.	20,273	20,274	20,275	20,279	20,276
ENC	49.90	49.87	49.86	49.88	49.83
CAI	0.166	0.166	0.167	0.167	0.166
CBI	−0.100	−0.101	−0.100	−0.100	−0.102
FOP	0.354	0.354	0.354	0.354	0.353
GC content (%)	0.382	0.382	0.382	0.382	0.382
GC3 content (%)	0.269	0.269	0.269	0.269	0.269

SC: synonymous codons; ENC: effective number of codons; CAI: codon adaptation index; CBI: codon bias index; FOP: frequency of optimal codons; GC content: G+C content of the genes; GC3 content: G+C content of synonymous third codons positions.

## Supplementary Materials

The following are available online at https://www.mdpi.com/2223-7747/9/4/543/s1, Table S1: The RSCU values of 64 codons in the five *Bupleurum* species, Table S2: 53 protein-coding genes (CDS) used in codon usage bias analysis and corresponding alignment length and GC content, Table S3: Complete plastid genomes of sixteen Apiaceae species and two Araliaceae species from GenBank, Figure S1: Molecular phylogenetic trees of 18 Apiaceae and 2 Araliaceae based on (A) complete plastid genomes; (B) LSC regions; (C) SSC regions; (D) IR regions; (E) single-copy CDS sequences; (F) ten high-variation regions.
